# The role of actigraphy in detecting and characterizing the early phases of Alzheimer’s disease

**DOI:** 10.1093/sleep/zsae076

**Published:** 2024-03-18

**Authors:** Joseph R Winer

**Affiliations:** Department of Neurology and Neurological Sciences, Stanford University, Stanford CA, USA

Alzheimer’s disease (AD) is defined by the presence of β-amyloid (Aβ) plaques and tau neurofibrillary tangles, which begin to aggregate in the brain many years before the onset of cognitive impairment [1, 2]. Advances in detecting AD neuropathology, including positron emission tomography (PET) radiotracers as well as cerebrospinal fluid and blood biomarkers, have greatly broadened our understanding of the “preclinical” phase of AD, between the first appearance of Aβ plaques and clinical decline. With the increasing availability and affordability of accurate disease biomarkers, there is now an opportunity to detect behavioral and cognitive changes in the presymptomatic phase of AD, before substantial neurodegeneration, when presumably the potential for early intervention or treatment is greatest.

It is generally understood that better sleep health in late life is associated with lower risk and/or slower progression of AD, a hypothesis supported by epidemiological findings [3, 4] as well as mechanistic studies in AD mouse models [5–7]. Combining AD biomarkers with wearable devices that track lifestyle factors such as sleep and physical activity has led to a better understanding of lifestyle-pathology interactions in preclinical AD as well as characterization of behavioral phenotypes. Cross-sectional studies using wrist-worn actigraphy recording in cognitively unimpaired older adults have shown worse sleep and greater rest-activity rhythm fragmentation are related to AD pathology [8–11]. Interestingly, greater night-to-night variability of sleep patterns is also associated with elevated AD pathology [12–14].

Accelerometers have been used to track sleep and physical activity for the past 40 years [15, 16]. While much about the technology has remained the same, there are two dimensions in which the actigraphy field is advancing. First, there has been an explosion of consumer devices that track 24-hour activity, and these devices are increasingly accepted by the sleep research community [17], allowing for real-world data collection on a scale not possible with “research-grade” devices alone. Second, the development of new techniques for analyzing these data provides opportunities to discover new signals in 24-hour behavior. In this issue of Sleep, Spira et al. demonstrate the potential of one such novel approach in identifying 24-hour activity differences in 82 cognitively impaired older adults with and without Aβ deposition (assessed by PET imaging) [18]. In addition to standard actigraphy metrics (which did not detect any differences between groups), the authors utilize function on scalar regression (FOSR). FOSR is a functional analysis method that effectively slices a mean 24-hour activity signal into 48 bins and statistically compares group differences within each 30-minute segment. This approach revealed that the Aβ-positive group was significantly more active than the Aβ-negative group between 1:00 pm and 3:30 pm. By calculating the standard deviation of each activity bin, the authors also showed that the Aβ-positive group had more regular activity during the early afternoon across the recording period.

The authors interpret these differences as reflecting circadian dysfunction in Aβ-positive individuals, possibly even an early manifestation of “sundowning.” Sundowning is typically defined as the exacerbation or onset of dementia-related neuropsychiatric symptoms (such as agitation, anxiety, or disorientation) in the late afternoon and evening [19–21] and is thought to impact about 20% of people living with AD [22]. Considering this definition, it seems unlikely that increased activity during the post-lunch period of 1:00–3:30 pm would represent sundowning-like behavior. More importantly, the implication that increased afternoon activity would be maladaptive is counterintuitive. Rather, it seems more plausible that increased activity in the early afternoon would represent greater physical activity, which would generally be considered beneficial in this population of healthy older adults.

A broader point for our field to consider is that research on sleep in presymptomatic AD has thus far primarily focused on how *deficits* in sleep and circadian health may be associated with the presence and progression of AD pathology. This emphasis on impairment makes sense because our understanding of sleep’s putative mechanistic role in AD has largely been based on rodent models, which have provided us with the bidirectional “vicious cycle” hypothesis [23, 24] whereby sleep disruption exacerbates pathological spread [5–7], and pathology further impairs sleep–wake cycles [25, 26]. While there is no denying the utility of such a framework, the converse may be equally if not more important. That is, are there 24-hour activity or sleep patterns that impart increased *resistance* to AD pathological progression, or *resilience* to cognitive decline in the presence of AD pathology? For instance, greater daytime activity levels are predictive of slower prospective cognitive decline [27], even in individuals who are Aβ-positive at baseline [28]. Widespread use of actigraphy has the potential to inform us how we might better time sleep and activity to prevent or slow AD, rather than indicate pathological associations with insufficient sleep and erratic activity patterns. Can techniques such as FOSR, as outlined by Spira et al., help us determine not just that more activity is better but that more activity at specific times of day (or more importantly, times of day relative to sleep schedules) can be protective?

With accurate and affordable blood biomarkers opening the field of AD research beyond those with access to PET imaging, there is enormous opportunity for the use of wearables to monitor real-life behavior and associate these behaviors with AD biomarkers at scale. Many large aging cohort studies have already implemented actigraphy [29, 30]. The expansion of this type of data collection will only be enhanced with the continued development of novel approaches such as FOSR and others [31–33] which can be refined through application to large longitudinal cohorts. We may come to understand the aspects of sleep and 24-hour activity patterns that are associated with accelerated progression of AD, and those that bestow resilience.
